# Death of a neonate with suspected coronavirus disease 2019 born to a mother with coronavirus disease 2019 in Iran: a case report

**DOI:** 10.1186/s13256-020-02519-1

**Published:** 2020-10-06

**Authors:** Tayebeh Rashidian, Nasibeh Sharifi, Azita Fathnezhad-Kazemi, Fatemeh Mirzamrajani, Sajad Nourollahi, Abas Ghaysouri

**Affiliations:** 1 Department of Obstetrics and Gynecology, Medical School, Ilam University of Medical Sciences, Ilam, Iran; 2 Department of Midwifery, School of Nursing & Midwifery, Ilam University of Medical Sciences, Ilam, Iran; 3 Department of Midwifery, Faculty of Nursing and Midwifery Islamic Azad University, Tabriz Branch, Tabriz, Iran; 4 Department of Pediatrics, Medical School, Ilam University of Medical Sciences, Ilam, Iran; 5 Department of Internal Medicine, Medical School, Ilam University of Medical Sciences, Ilam, Iran

**Keywords:** Coronavirus 19, Novel coronavirus, Infant death, Mother-to-child transmissions

## Abstract

**Introduction:**

A novel coronavirus named severe acute respiratory syndrome coronavirus 2, was first reported in Wuhan, China, in December 2019. The virus, known as COVID-19, is recognized as a potentially life-threatening disease by causing severe respiratory disease. Since this virus has not previously been detected in humans, there is a paucity of information regarding its effects on humans. In addition, only limited or no information exists about its impact during pregnancy.

**Case presentation:**

In the present case study, we report the death of a neonate born to a 32-year-old mother with coronavirus disease 2019 in Ilam, Iran, with Kurdish ethnicity. We report the infection and death of a neonate in Iran with a chest X-ray (CXR) marked abnormality 2 hours after birth demonstrating coronavirus disease 2019 disease. The neonate was born by elective cesarean section, the fetal health was assessed using fetal heart rate and a non-stress test before the birth, and there was no evidence of fetal distress. All the above-mentioned facts and radiographic abnormalities suggested that coronavirus disease 2019 is involved.

**Conclusions:**

In this case study, we report the death of a neonate born to a mother with coronavirus disease 2019, 11 hours after birth. There is a paucity of data on the vertical transmission and the adverse maternal-fetal consequences of this disease, so vertical transmission from mother to child remains to be confirmed.

## Introduction

The spread of the novel coronavirus, scientifically known as severe acute respiratory syndrome coronavirus 2 (SARS-Cov-2), a potentially life-threatening respiratory disease, has led to a major public health problem and a threat to the health of millions of people worldwide [1, 2]. The World Health Organization (WHO) issued a statement on January 11, 2019, declaring the novel coronavirus (2019-nCoV) the sixth public health emergency worldwide [3]. The increased incidence and global spread of this virus has caused great concern and panic among people all over the world [4, 5].

According to the latest meta-analysis of 50,466 patients with SARS-Cov-2, known as COVID-19, the virus has led to the mortality rate of 4.3% [1, 6, 7]. The prevalence and mortality due to outbreaks of novel coronavirus disease 2019 is very high in Iran [7, 8]. Elderly people with underlying health conditions, people with weakened immune systems, and pregnant women are at greater risk to develop COVID-19. Pregnant women are at higher risk to develop severe cases and complications of serious illness from COVID-19 due to changes in the immune and pulmonary systems during pregnancy [9]. Therefore, the prevention and control of COVID-19 infection among pregnant women and the potential risk of vertical transmission have become a major concern. Due to the new coronavirus epidemic, limited studies have been conducted on the effect of COVID-19 infection on mother and fetus [9, 10]. Although the obtained information and data on the other highly pathogenic coronaviruses, such as acute respiratory syndrome (SARS) and Middle East respiratory syndrome (MERS) were indicative of serious complications in the neonates born to these mothers [11, 12]. Nonetheless, the clinical characteristics and vertical transmission potential of COVID-19 pneumonia in pregnant women are unknown [13]. The present case study reported the infection and death of a neonate in Iran with a chest X-ray (CXR) marked abnormality 2 hours after birth demonstrating COVID-19.

## Case presentation

The mother of the deceased neonate is a 32-year-old pregnant woman (G1P1) living in Ivan in Ilam province, with Kurdish ethnicity. Ivan is one of the cities with a high prevalence of the COVID-19 in Ilam (a province in southwestern Iran). The mother had no history of any serious diseases (for example, blood pressure disorders, autoimmune disease, diabetes, or thyroid disorder), and no history of substance abuse (for example, smoking, alcohol, or drug addiction).

There were not any pregnancy complications, such as placental abruption, rupture of membranes for more than 18 hours, meconium passage, and impaired fetal heart rate (FHR). It is notable that she had only taken supplements, such as iron and multivitamins during pregnancy. Routine ultrasound screening for fetal abnormalities was normal. Based on 11-week ultrasound, the mother was admitted at 39 weeks of gestation to undergo elective C-section at Zagros Hospital (a semi-private hospital) in Ilam at 8:00 a.m. on March 10, 2020.

According to the last ultrasound, the amniotic fluid volume was normal and the non-stress test (NST) was reactive at the admission to hospital. Also, the mother’s vital signs, including her body temperature, pulse rate, respiratory rate, and blood pressure were normal. According to the mother’s self-reports during hospitalization, she had no COVID-19 symptoms. At 9:30 a.m. on March 10, half an hour after hospitalization, the mother was sent to the operating room in a good general condition and a fetal heart rate of 145 (Table 1).

At 10 a.m., the seemingly healthy neonate was born in cephalic position by cesarean delivery. The 1- and 5-minute Apgar scores were 9 and 10, respectively. The newborn’s birth weight, head circumference, and height were 3545 grams, 34 cm and 49 cm, respectively. No abnormalities were detected on the initial examination after birth and the newborn was delivered to the neonatal ward in a stable condition (Table 2).

At 11:30 a.m., half an hour after birth, the neonate was visited by a neonatal specialist at the Zagros Hospital in Ilam with grunting and respiratory distress. In accordance with the doctor’s instructions, he was given oxygen therapy by oxygen hood. The neonate’s initial vital signs were as follows: respiration rate (RR) of 67, temperature (T) of 37 °C, pulse rate (PR) of 128, oxygen saturation (SpO2) of 97%, and with a clear grunting sound. Serum therapy was started and the neonate’s blood glucose level was checked (a blood sugar level of 72). At 12:10 p.m., the neonate was revisited and transferred to the neonatal intensive care unit (NICU) of Ayatollah Taleghani Hospital on the newborn’s doctor’s order due to his sustained problem.

After coordination with the officials of the hospital, the neonate was transferred to Taleghani Hospital at 1:00 p.m. with respiratory distress, he was cyanotic, his oxygen saturation had fallen, and he was bleeding. At 2:00 p.m., the neonate was admitted to the NICU of Taleghani Hospital with moaning and respiratory distress. Upon arrival at the hospital, the newborn had a marked decrease in consciousness level and his vital signs were as follows: BP of 40.23, PR of 145, RR of 33, SpO2 of 34%, and T of 36.5 °C. When entering the NICU, the newborn was cyanotic and had irregular respiration and low oxygen saturation.

**Fig. 1:**
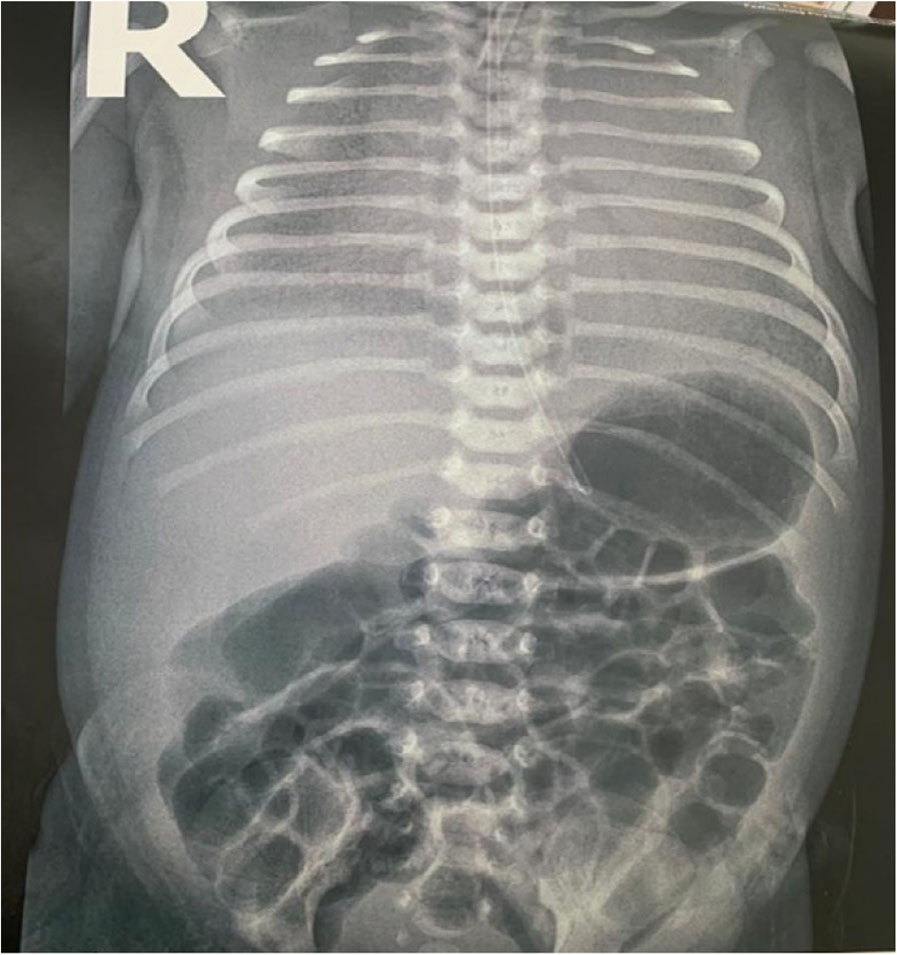
Newborn chest radiography

He was immediately intubated in NICU and ventilated with an Ambu neonatal self-inflating bag and received three doses of intravenous epinephrine, emergency fresh frozen plasma, emergency palliative care, three vials of surfactants, 3 mg of vitamin K, and emergency cardiac counseling. Clear nasal and tracheal tube bleeding was reported; therefore, a chest X-ray (CXR) was performed, and the appearance of both lungs was quite similar to signs of coronavirus disease 19 (COVID-19) on the emergency CXR (Fig. 1).

**Table 1:** Clinical features of mothers with 2019-nCoV infection

**Age (years)**	32
**Maternal history**	G1P1
**First symptom**	Dry coughs
**Intrauterine fetal distress**	No
**Vital signs**	Normal
**Other medical histories during pregnancy**	No
**Delivery type**	Elective Cesarean section
**Premature rupture of membranes**	No
**Placenta**	Normal
**Amniotic fluid**	Normal

**Table 2:** Information of neonates born to mothers with confirmed 2019-nCoV infection

**Gender**	Male
**Gestational age (weeks)**	39 weeks
**Birth weight (g)**	3545
**Pregnancy**	Singleton
**Apgar score 1-min**	9–10
**First symptom**	Grunting and respiratory distress
**Other symptoms**	Respiratory distress, cyanotic, fall of oxygen saturation, and bleeding
**Outcome**	Died

The summary of the neonate’s laboratory findings is as follows: a white blood cell count of 15.7, hemoglobin (Hb) level of 14.8, platelet blood count of 181, prothrombin time of 17.9, partial thromboplastin time of 56, an international normalized ratio of 1.8, a pH level of 7.1, partial pressure of carbon dioxide of 67, bicarbonate (HCo3) level of 13.7, creatinine level of 1.1, sodium level of 134, and urea level of 12. A blood culture was also negative.

At 7:00 p.m., the newborn experienced decreased oxygen saturation and bleeding, and at 9:00 p.m., cardiopulmonary resuscitation was performed for 30 minutes due to the fall of his blood saturation. The newborn did not respond positively to any conducted treatment for 6 hours after hospitalization and died at 9:30 p.m.

Following a clinical deterioration in the neonate and the abnormal CXR, the mother was questioned again about symptoms of COVID-19. In addition, she underwent a blood test and a CXR. According to the mother, she only had a single dry cough within a family history of coronavirus in her cousin, which resulted in his death. The mother’s test results were prepared at 3:56 p.m., which were as follows.

A white blood cell (WBC) count of 12.7, Hb level of 12.1, hematocrit level of 35.5, erythrocyte sedimentation rate of 40 mm/hour, C-reactive protein (CRP: positive [2+]). Immediately after examining the test results, due to the high level of CRP and the probability of COVID-19 infection, the throat swab samples of the mother were tested by reverse transcription polymerase chain reaction (RT-PCR) and positive results confirmed that she was infected with 2019-nCoV.

It should be noted that, the neonatal pharyngeal swabs (RT-PCR) tested negative for 2019-nCoV, but due to the death of the baby, there was no opportunity for resampling.

## Discussion

Over the past few months, the COVID-19 pandemic has caused significant concern around the world due to its rapid spread [14, 15]. During pregnancy, women are exposed to respiratory pathogens and severe pneumonia due to weakened immune systems and physiological changes during this period, such as increased heart rate, stroke volume, increased diaphragm level, increased oxygen consumption, and mucosal edema, as well as decreased lung capacity [13].

According to the results of clinical studies, patients with COVID-19 infection during pregnancy are similar to non-pregnant people with infection [13]. The results of some studies suggest that pregnant women with respiratory infections with different viruses have more adverse maternal and fetal consequences, as compared to nonaffected women [16, 17]. In a study conducted in Hong Kong, maternal infection with SARS-CoV increased the risk of adverse outcomes, such as miscarriage, preterm delivery, and maternal death [18].

Other findings have suggested that COVID-19 may cause adverse effects, such as fetal distress, preterm delivery, respiratory distress, thrombocytopenia with abnormal liver function, and even death [19]. Evidence of vertical transmission been reported in other respiratory viruses, such as H1N1 and the respiratory syncytial virus [20].

In the present study, the neonate was born to an infected mother without any clinical signs of coronavirus disease (she only had an occasional single dry cough). The neonate’s clinical manifestations were mild in the early hours of birth; nonetheless, he developed severe respiratory distress and cyanosis a few hours after birth.

The question is the possibility of vertical transfer in mothers with mild symptoms who test positive for coronavirus during this pandemic.

The newborn, whose condition worsened, was examined for congenital anomalies and underwent complete blood tests, blood culture, and PCR. In one assessment, the newborn’s nucleic acid detection results were negative, which does not support the diagnosis of intrauterine transmission. However, the possibility of vertical intrauterine transmission of SARS-CoV-2 is not ruled out. He also underwent CXR in which his lung presentation was similar to people with coronavirus disease. Negative test results may be due to some factors, such as insufficient viral load or high false-negative test results.

The neonate was born by elective cesarean section, and the fetal health assessment was performed before the birth by examining fetal heart rate and his NST, demonstrating no evidence of fetal distress. Moreover, the postpartum evaluation also rejected any cardiovascular diseases and abnormalities. All the above-mentioned reasons along with the X-ray abnormalities raised the possibility that the newborn may have COVID-19. Furthermore, vertical transmission is not beyond the bounds of possibility based on the results of other studies in other countries.

In a study by Moeindarbary *et al.*, researchers reported infants with coronavirus infection and that they had worse clinical condition than their mothers. The severity of pneumonia and the degree of lung involvement in these infants were not related to the stage and severity of the disease in infected mothers [21]. Chen *et al.*’s study in Wuhan also reported vertical transmission between pregnant women with COVID-19 and their infants [22]. According to research results, COVID-19 gravely damages leukocytes, and could lead to multiple organ damage along with the respiratory system [23]. As reported in China, the risk of transmission cannot be ruled out [1, 19, 24]. The results of a study carried out by Zhu *et al.* who analyzed the clinical analysis of 10 neonates born to mothers with COVID-19 suggested that coronavirus infection during pregnancy may exert adverse effects on newborns. It can cause serious problems, such as fetal distress, preterm delivery, respiratory distress, thrombocytopenia with abnormal liver function, and even death. However, vertical transmission of this virus has not yet been confirmed [19]. Previous epidemics of viral infections have reported poor pregnancy outcomes, including maternal mortality, mother-child transmission of the virus, perinatal infections, and fetal death [13, 24]. The existing data on the transmission of virus to neonates born to mothers with COVID-19 are not only insufficient, but also contradictory. In this regard, the results of some studies indicated high Apgar score, the absence of asphyxia, and neonatal infection, and there exists no report on the vertical transmission of this virus. Some studies have suggested cord blood and amniotic fluid sampling, stools from infants, and placental tissue examination to determine placental inflammation due to viral infection [22, 25].

According to the results of sonography (scan anomaly), the baby did not have any abnormalities and at birth, he was thoroughly examined by a neonatal specialist, but no abnormalities were observed. For other differential diagnoses, transient metabolic diseases and transient tachypnea are also ruled out, because metabolic disease usually appears a few days after birth, but the baby had respiratory symptoms and so on at birth and, in the case of transient tachypnea, this problem does not cause the death of the baby, and this baby received all the treatment at birth and yet died after 6 hours.

## Conclusions

In this case study, we report the death of a neonate born to a mother with COVID-19, 11 hours after birth. There is a paucity of data on the vertical transmission and the adverse maternal-fetal consequences of this disease. It could be due to the novelty of the disease and the lack of similar cases, which requires more comprehensive studies and further evaluation of affected mothers.

## Data Availability

All data and material collected during this study are available from the corresponding author upon reasonable request.
